# Bis(μ-4-methyl­benzoato-κ^2^ *O*:*O*′)bis­[aqua­(4-methyl­benzoato-κ^2^ *O*,O′)zinc(II)]–bis­(μ-4-methyl­benzoato-κ^2^ *O*:*O*′)bis­[(4-methyl­benzoato-κ*O*)(nicotinamide-κ*N*)zinc(II)]–water (1/1/2)

**DOI:** 10.1107/S1600536810022476

**Published:** 2010-06-26

**Authors:** Tuncer Hökelek, Emel Ermiş, Barış Tercan, Efdal Çimen, Hacali Necefoğlu

**Affiliations:** a Department of Physics, Hacettepe University, 06800 Beytepe, Ankara, Turkey; b Department of Chemistry, Faculty of Science, Anadolu University, 26470 Yenibağlar, Eskişehir, Turkey; c Department of Physics, Karabük University, 78050 Karabük, Turkey; d Department of Chemistry, Kafkas University, 63100 Kars, Turkey

## Abstract

The crystal structure of the title compound, [Zn_2_(C_8_H_7_O_2_)_4_(H_2_O)_2_]·[Zn_2_(C_8_H_7_O_2_)_4_(C_6_H_6_N_2_O)_2_]·2H_2_O, consists of two kinds of dinuclear Zn^II^ complexes (complex *A* and complex *B*) and uncoordinated water mol­ecules. In complex *A*, [Zn_2_(C_8_H_7_O_2_)_4_(H_2_O)_2_], each Zn cation is chelated by a 4-methyl­benzoate (PMB) anion and coordinated by a water mol­ecule, and is further bridged by two PMB anions in a trigonal-bipyramidal geometry. In complex *B*, [Zn_2_(C_8_H_7_O_2_)_4_(C_6_H_6_N_2_O)_2_], each Zn^II^ cation is coordinated by a monodentate PMB anion and one nicotinamide (NA) ligand, and is further bridged by two PMB anions in a tetra­hedral geometry. Weak intra-mol­ecular π–π stacking between adjacent benzene rings is observed in complex *B*, the centroid–centroid distance being 3.710 (2) Å. Extensive O—H⋯O and N—H⋯O hydrogen bonding and weak C—H⋯O hydrogen bonding is present in the crystal structure. The crystal studied was a racemic twin; the minor twin component refined to 38%.

## Related literature

For related structures, see: Greenaway *et al.* (1984 ▶); Hökelek & Necefoğlu (1996 ▶); Hökelek *et al.* (2009*a* ▶,*b* ▶,*c* ▶,*d* ▶).
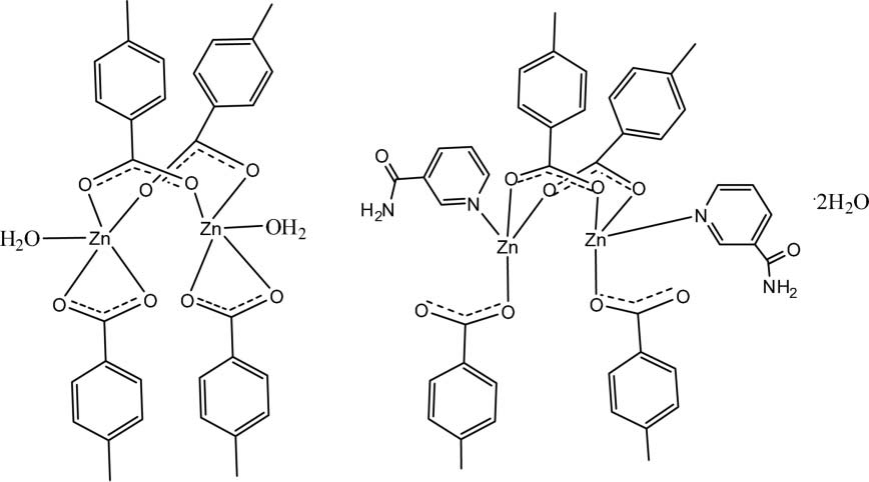


## Experimental

### 

#### Crystal data

[Zn_2_(C_8_H_7_O_2_)_4_(H_2_O)_2_]·[Zn_2_(C_8_H_7_O_2_)_4_(C_6_H_6_N_2_O)_2_]·2H_2_O
*M* *_r_* = 1658.97
Monoclinic, 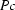

*a* = 19.7038 (3) Å
*b* = 12.2884 (2) Å
*c* = 15.4477 (3) Å
β = 98.708 (1)°
*V* = 3697.21 (11) Å^3^
*Z* = 2
Mo *K*α radiation
μ = 1.36 mm^−1^
*T* = 100 K
0.35 × 0.25 × 0.15 mm

#### Data collection

Bruker Kappa APEXII CCD area-detector diffractometer
Absorption correction: multi-scan (*SADABS*; Bruker, 2005 ▶) *T*_min_ = 0.718, *T*_max_ = 0.863
35979 measured reflections
16444 independent reflections
14033 reflections with *I* > 2σ(*I*)
*R*_int_ = 0.044

#### Refinement

*R*[*F* ^2^ > 2σ(*F* ^2^)] = 0.040
*wR*(*F* ^2^) = 0.080
*S* = 1.01
16444 reflections
964 parameters
2 restraints
H-atom parameters constrained
Δρ_max_ = 0.38 e Å^−3^
Δρ_min_ = −0.52 e Å^−3^
Absolute structure: Flack (1983 ▶), 7229 Friedel pairs
Flack parameter: 0.382 (7)

### 

Data collection: *APEX2* (Bruker, 2007 ▶); cell refinement: *SAINT* (Bruker, 2007 ▶); data reduction: *SAINT*; program(s) used to solve structure: *SHELXS97* (Sheldrick, 2008 ▶); program(s) used to refine structure: *SHELXL97* (Sheldrick, 2008 ▶); molecular graphics: *Mercury* (Macrae *et al.*, 2006 ▶); software used to prepare material for publication: *WinGX* (Farrugia, 1999 ▶) and *PLATON* (Spek, 2009 ▶).

## Supplementary Material

Crystal structure: contains datablocks I, global. DOI: 10.1107/S1600536810022476/xu2762sup1.cif

Structure factors: contains datablocks I. DOI: 10.1107/S1600536810022476/xu2762Isup2.hkl

Additional supplementary materials:  crystallographic information; 3D view; checkCIF report

## Figures and Tables

**Table 1:** Selected bond lengths (Å)

Zn1—O1	1.964 (2)
Zn1—O3	1.939 (3)
Zn1—O7	2.505 (3)
Zn1—O8	1.975 (2)
Zn1—O9	1.966 (2)
Zn2—O2	1.942 (3)
Zn2—O4	1.967 (2)
Zn2—O5	2.467 (3)
Zn2—O6	1.989 (3)
Zn2—O10	1.994 (2)
Zn3—O11	1.926 (2)
Zn3—O13	1.967 (3)
Zn3—O16	1.932 (2)
Zn3—N1	2.047 (3)
Zn4—O12	1.968 (3)
Zn4—O14	1.924 (2)
Zn4—O17	1.940 (2)
Zn4—N3	2.036 (3)

**Table 2:** Hydrogen-bond geometry (Å, °)

*D*—H⋯*A*	*D*—H	H⋯*A*	*D*⋯*A*	*D*—H⋯*A*
N2—H2*A*⋯O5^i^	0.88	2.29	3.161 (4)	173
N2—H2*B*⋯O21	0.88	2.06	2.918 (4)	165
N4—H4*A*⋯O7^ii^	0.88	2.19	3.061 (4)	168
N4—H4*B*⋯O22	0.88	2.08	2.939 (5)	166
O9—H91⋯O5^iii^	0.97	1.65	2.602 (3)	166
O9—H92⋯O19^iv^	0.97	1.68	2.644 (4)	172
O10—H101⋯O20^v^	0.97	1.67	2.639 (4)	176
O10—H102⋯O7^vi^	0.97	1.78	2.649 (3)	147
O21—H211⋯O15	0.97	1.94	2.869 (4)	159
O21—H212⋯O19^vii^	0.86	2.29	3.138 (4)	171
O22—H221⋯O20^viii^	0.97	2.23	3.164 (4)	160
O22—H222⋯O18	0.97	1.90	2.802 (5)	152
C65—H65⋯O21	0.95	2.37	3.282 (4)	161
C71—H71⋯O22	0.95	2.33	3.256 (5)	165

Symmetry codes: (i) 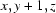; (ii) 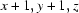; (iii) 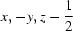; (iv) 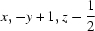; (v) 
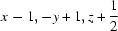
; (vi) 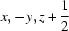; (vii) 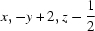; (viii) 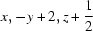.

**Fig. 1.**
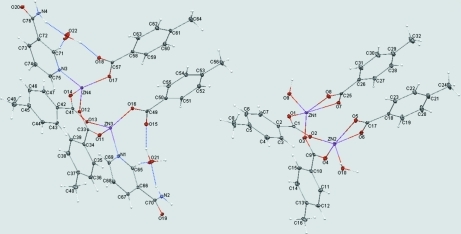
The molecular structure of the title molecule with the atom-numbering scheme. Displacement ellipsoids are drawn at the 20% probability level. Hydrogen bonds are shown as dashed lines.

## supplementary crystallographic information

### Comment

As part of our ongoing study on transition metal complexes of benzoate and nicotinamide, herein we report the synthesis and the structure of the title cocrystal diaquabis(µ-4-methylbenzoato-κ^2^*O*:*O*')- bis(4-methylbenzoato-κ^2^*O*:*O*')dizinc(II), (A), and bis(nicotinamide-κ*N*')- bis(µ-4-methylbenzoato-κ^2^*O*:*O*')bis(4-methylbenzoato-κ*O*)dizinc(II) dihydrate, (B).

The components of the title compound, [Zn_2_(C_8_H_7_O_2_)_4_(H_2_O)_2_], (A), and [Zn_2_(C_8_H_7_O_2_)_4_(C_6_H_6_N_2_O)_2_].2(H_2_O), (B), are binuclear complexes. In the complex A, each Zn cation is chelated by a 4-methylbenzoate (PMB) anion and coordinated by a water molecule, and is further bridged by two PMB anions with a trigonal bipyramidal geometry. In the complex B, each Zn^II^ cation is coordinated by a monodentate PMB anion and one nicotinamide (NA) ligand, and is further bridged by two PMB anions with a tetrahedral geometry. The intramolecular O—H···O, N—H···O and C—H···O hydrogen bonds (Table 2) link the two uncoordinated water molecules to the O, N and C atoms of the carboxylate and NA groups, respectively (Fig. 1).

The average Zn—O bond lengths (Table 1) are 2.071 (3) and 1.942 (3) Å for (A) and (B), respectively, and the Zn atoms are displaced out of the least-squares planes of the carboxylate groups: Zn1 atom for (O1/C1/O2), (O3/C9/O4) and (O7/C25/O8) by 0.0892 (4), -0.0858 (4) and -0.2690 (4) Å, respectively, Zn2 atom for (O1/C1/O2), (O3/C9/O4) and (O5/C17/O6) by -0.0702 (4), 0.0213 (4) and -0.2301 (4) Å, respectively, Zn3 atom for (O11/C33/O12), (O13/C41/O14) and (O15/C49/O16) by -0.1442 (4), -0.0176 (4) and 0.0844 (3) Å, respectively, and Zn4 atom for (O11/C33/O12), (O13/C41/O14) and (O17/C57/O18) by 0.0461 (4), 0.1211 (4) and -0.0012 (3) Å, respectively. In (A), the O7—Zn1—O8 and O5—Zn2—O6 angles are 57.37 (10) and 58.03 (10) °, respectively. The corresponding O—M—O (where *M* is a metal) angles are 52.91 (4)° and 53.96 (4)° in [Cd(C_8_H_5_O_3_)_2_(C_6_H_6_N_2_O)_2_(H_2_O)].H_2_O (Hökelek *et al.*, 2009a), 60.70 (4)° in [Co(C_9_H_10_NO_2_)_2_(C_6_H_6_N_2_O)(H_2_O)_2_] (Hökelek *et al.*, 2009b), 58.45 (9)° in [Mn(C_9_H_10_NO_2_)_2_(C_6_H_6_N_2_O)(H_2_O)_2_] (Hökelek *et al.*, 2009c), 60.03 (6)° in [Zn(C_8_H_8_NO_2_)_2_(C_6_H_6_N_2_O)_2_].H_2_O (Hökelek *et al.*, 2009d), 58.3 (3)° in [Zn_2_(DENA)_2_(C_7_H_5_O_3_)_4_].2H_2_O (Hökelek & Necefoğlu, 1996) and 55.2 (1)° in [Cu(Asp)_2_(py)_2_] (where Asp is acetylsalicylate and py is pyridine) (Greenaway *et al.*, 1984).

The dihedral angles between the planar carboxylate groups and the adjacent benzene rings A (C2—C7), B (C10—C15), C (C18—C23), D (C26—C31), G (C34—C39), H (C42—C47), I (C50—C55) and J (C58—C63) are 4.44 (26), 1.40 (21), 5.40 (15), 8.20 (14), 3.33 (26), 10.45 (26), 9.93 (10) and 9.86 (11) °, respectively, while those between rings A, B, C, D, E (Zn1/O7/O8/C25), F (Zn2/O5/O6/C17) and G, H, I, J, K (N1/C65—C69), *L* (N3/C71—C75) are A/B = 63.10 (12), A/C = 77.01 (12), A/D = 82.24 (13), B/C = 78.45 (12), B/D = 86.45 (14), C/D = 8.23 (13), E/F = 6.17 (9) ° and G/H = 62.85 (10), G/I = 79.23 (12), G/J = 78.86 (12), H/I = 81.13 (12), H/J = 80.21 (11), I/J = 1.32 (10), K/*L* = 65.25 (10) °.

In the crystal structure, extensive O—H···O, N—H···O hydrogen bonding and weak C—H···O hydrogen bonding (Table 2) are present, in which they may be effective in the stabilization of the structure. The π–π contact between the benzene rings, *Cg*9—*Cg*10, where *Cg*9 and *Cg*10 are the centroids of the rings  I (C50—C55) and J (C58—C33), respectively] may further stabilize the structure, with centroid-centroid distance of 3.710 (2) Å.

### Experimental

The title compound was prepared by the reaction of ZnSO_4_.H_2_O (0.90 g, 5 mmol) in H_2_O (200 ml) and NA (1.22 g, 10 mmol) in H_2_O (10 ml) with sodium 4-methylbenzoate (1.58 g, 10 mmol) in H_2_O (150 ml). The mixture was filtered and set aside to crystallize at ambient temperature for one week, giving colorless single crystals.

### Refinement

Water H atoms were placed in chemical sensitive positions and refined in a riding mode with U_iso_(H) = 1.2U_eq_(O). Methyl H atoms were placed in calculated positions with C—H = 0.98 Å, and torsion angles were refined to fit the electron density, U_iso_(H) = 1.5U_eq_(C). The remaining H atoms were positioned geometrically with N—H = 0.88 Å and C—H = 0.95 Å, and constrained to ride on their parent atoms with U_iso_(H) = 1.2U_eq_(C,N).

### Crystal data

**Table:**

[Zn_2_(C_8_H_7_O_2_)_4_(H_2_O)_2_]·[Zn_2_(C_8_H_7_O_2_)_4_(C_6_H_6_N_2_O)_2_]·2H_2_O	*F*(000) = 1712
*M**_r_* = 1658.97	*D*_x_ = 1.490 Mg m^−^^3^
Monoclinic, *P**c*	Mo *K*α radiation, λ = 0.71073 Å
Hall symbol: P -2yc	Cell parameters from 7336 reflections
*a* = 19.7038 (3) Å	θ = 2.5–25.5°
*b* = 12.2884 (2) Å	µ = 1.36 mm^−^^1^
*c* = 15.4477 (3) Å	*T* = 100 K
β = 98.708 (1)°	Block, colorless
*V* = 3697.21 (11) Å^3^	0.35 × 0.25 × 0.15 mm
*Z* = 2	

### Data collection

**Table:**

Bruker Kappa APEXII CCD area-detector diffractometer	16444 independent reflections
Radiation source: fine-focus sealed tube	14033 reflections with *I* > 2σ(*I*)
graphite	*R*_int_ = 0.044
φ and ω scans	θ_max_ = 28.3°, θ_min_ = 1.1°
Absorption correction: multi-scan (*SADABS*; Bruker, 2005)	*h* = −26→23
*T*_min_ = 0.718, *T*_max_ = 0.863	*k* = −16→16
35979 measured reflections	*l* = −20→20

### Refinement

**Table:**

Refinement on *F*^2^	Secondary atom site location: difference Fourier map
Least-squares matrix: full	Hydrogen site location: inferred from neighbouring sites
*R*[*F*^2^ > 2σ(*F*^2^)] = 0.040	H-atom parameters constrained
*wR*(*F*^2^) = 0.080	*w* = 1/[σ^2^(*F*_o_^2^) + (0.0318*P*)^2^] where *P* = (*F*_o_^2^ + 2*F*_c_^2^)/3
*S* = 1.01	(Δ/σ)_max_ = 0.004
16444 reflections	Δρ_max_ = 0.38 e Å^−^^3^
964 parameters	Δρ_min_ = −0.52 e Å^−^^3^
2 restraints	Absolute structure: Flack (1983), 7229 Friedel pairs
Primary atom site location: structure-invariant direct methods	Flack parameter: 0.382 (7)

### Special details

**Table:**

Geometry. All esds (except the esd in the dihedral angle between two l.s. planes) are estimated using the full covariance matrix. The cell esds are taken into account individually in the estimation of esds in distances, angles and torsion angles; correlations between esds in cell parameters are only used when they are defined by crystal symmetry. An approximate (isotropic) treatment of cell esds is used for estimating esds involving l.s. planes.
Refinement. Refinement of *F*^2^ against ALL reflections. The weighted *R*-factor *wR* and goodness of fit *S* are based on *F*^2^, conventional *R*-factors *R* are based on *F*, with *F* set to zero for negative *F*^2^. The threshold expression of *F*^2^ > σ(*F*^2^) is used only for calculating *R*-factors(gt) *etc*. and is not relevant to the choice of reflections for refinement. *R*-factors based on *F*^2^ are statistically about twice as large as those based on *F*, and *R*- factors based on ALL data will be even larger.

### Fractional atomic coordinates and isotropic or equivalent isotropic displacement parameters (Å^2^)

**Table:**

	*x*	*y*	*z*	*U*_iso_*/*U*_eq_	
Zn1	0.28355 (2)	0.10592 (3)	0.15711 (2)	0.01471 (9)	
Zn2	0.20670 (2)	0.07066 (3)	0.32791 (2)	0.01696 (10)	
Zn3	0.69302 (2)	0.99800 (3)	0.33479 (2)	0.01354 (10)	
Zn4	0.79811 (2)	1.00907 (3)	0.16901 (2)	0.01335 (10)	
N1	0.63153 (16)	1.0446 (2)	0.42375 (17)	0.0155 (7)	
N2	0.41642 (17)	0.9780 (2)	0.39318 (18)	0.0175 (7)	
H2A	0.3733	0.9576	0.3894	0.021*	
H2B	0.4376	0.9734	0.3471	0.021*	
N3	0.85772 (15)	1.0568 (2)	0.07915 (17)	0.0137 (6)	
N4	1.07081 (17)	0.9790 (2)	0.0974 (2)	0.0201 (7)	
H4A	1.1138	0.9580	0.1001	0.024*	
H4B	1.0497	0.9714	0.1433	0.024*	
O1	0.34255 (14)	0.18920 (19)	0.24754 (14)	0.0201 (6)	
O2	0.28988 (14)	0.1544 (2)	0.36336 (15)	0.0205 (6)	
O3	0.19960 (13)	0.1879 (2)	0.12372 (14)	0.0200 (6)	
O4	0.14732 (14)	0.1571 (2)	0.23982 (15)	0.0220 (6)	
O5	0.26286 (15)	−0.0934 (2)	0.39755 (15)	0.0205 (6)	
O6	0.17315 (14)	−0.0784 (2)	0.29432 (16)	0.0205 (6)	
O7	0.22349 (14)	−0.0618 (2)	0.09298 (15)	0.0192 (6)	
O8	0.30657 (14)	−0.0412 (2)	0.20415 (15)	0.0215 (6)	
O9	0.32937 (13)	0.1279 (2)	0.05389 (14)	0.0175 (6)	
H91	0.3002	0.1244	−0.0029	0.021*	
H92	0.3650	0.0733	0.0528	0.021*	
O10	0.16159 (13)	0.1037 (2)	0.43203 (15)	0.0194 (6)	
H101	0.1272	0.0502	0.4417	0.023*	
H102	0.1988	0.0972	0.4810	0.023*	
O11	0.64993 (14)	1.0712 (2)	0.23049 (15)	0.0195 (6)	
O12	0.71470 (14)	1.09187 (19)	0.12404 (15)	0.0173 (6)	
O13	0.77572 (14)	1.08106 (18)	0.38194 (15)	0.0162 (6)	
O14	0.84098 (14)	1.0780 (2)	0.27519 (14)	0.0182 (6)	
O15	0.60792 (14)	0.8331 (2)	0.27054 (15)	0.0204 (6)	
O16	0.71267 (14)	0.84434 (19)	0.34807 (15)	0.0170 (6)	
O17	0.77405 (14)	0.85598 (18)	0.15918 (15)	0.0160 (6)	
O18	0.87894 (14)	0.8431 (2)	0.23425 (16)	0.0205 (6)	
O19	0.42153 (14)	1.0236 (2)	0.53595 (15)	0.0186 (6)	
O20	1.06431 (14)	1.0350 (2)	−0.04234 (16)	0.0233 (6)	
O21	0.47309 (16)	0.9262 (3)	0.23420 (17)	0.0376 (9)	
H211	0.5212	0.9078	0.2363	0.045*	
H212	0.4546	0.9419	0.1818	0.045*	
O22	1.01450 (19)	0.9193 (3)	0.2565 (2)	0.0570 (12)	
H221	1.0405	0.9309	0.3143	0.068*	
H222	0.9737	0.8782	0.2648	0.068*	
C1	0.3372 (2)	0.1966 (3)	0.3273 (2)	0.0161 (8)	
C2	0.39105 (19)	0.2606 (3)	0.3843 (2)	0.0151 (8)	
C3	0.3864 (2)	0.2793 (3)	0.4718 (2)	0.0207 (9)	
H3	0.3485	0.2516	0.4963	0.025*	
C4	0.4373 (2)	0.3387 (3)	0.5235 (2)	0.0264 (9)	
H4	0.4338	0.3515	0.5833	0.032*	
C5	0.4930 (2)	0.3792 (3)	0.4892 (2)	0.0277 (10)	
C6	0.4968 (2)	0.3615 (3)	0.4017 (2)	0.0284 (9)	
H6	0.5345	0.3897	0.3771	0.034*	
C7	0.4462 (2)	0.3030 (3)	0.3495 (2)	0.0242 (9)	
H7	0.4494	0.2919	0.2894	0.029*	
C8	0.5502 (2)	0.4410 (4)	0.5459 (3)	0.0426 (12)	
H8A	0.5738	0.4881	0.5088	0.064*	
H8B	0.5830	0.3891	0.5769	0.064*	
H8C	0.5306	0.4856	0.5886	0.064*	
C9	0.1499 (2)	0.1971 (3)	0.1652 (2)	0.0145 (8)	
C10	0.0896 (2)	0.2605 (3)	0.1232 (2)	0.0159 (8)	
C11	0.03233 (19)	0.2751 (3)	0.1654 (2)	0.0205 (8)	
H11	0.0323	0.2449	0.2220	0.025*	
C12	−0.0243 (2)	0.3327 (3)	0.1262 (2)	0.0246 (9)	
H12	−0.0624	0.3419	0.1565	0.029*	
C13	−0.02635 (19)	0.3774 (3)	0.0431 (2)	0.0206 (8)	
C14	0.0302 (2)	0.3623 (3)	0.0009 (2)	0.0261 (9)	
H14	0.0297	0.3917	−0.0561	0.031*	
C15	0.08771 (19)	0.3052 (3)	0.0402 (2)	0.0190 (8)	
H15	0.1260	0.2967	0.0101	0.023*	
C16	−0.0885 (2)	0.4377 (3)	−0.0005 (3)	0.0328 (10)	
H16A	−0.1135	0.4681	0.0442	0.049*	
H16B	−0.1185	0.3876	−0.0380	0.049*	
H16C	−0.0741	0.4969	−0.0362	0.049*	
C17	0.2132 (2)	−0.1365 (3)	0.3483 (2)	0.0162 (8)	
C18	0.1993 (2)	−0.2550 (3)	0.3528 (2)	0.0188 (8)	
C19	0.1421 (2)	−0.3021 (3)	0.3038 (2)	0.0252 (9)	
H19	0.1100	−0.2575	0.2678	0.030*	
C20	0.1312 (2)	−0.4126 (3)	0.3065 (2)	0.0355 (11)	
H20	0.0916	−0.4429	0.2724	0.043*	
C21	0.1768 (2)	−0.4807 (3)	0.3578 (2)	0.0327 (10)	
C22	0.2338 (2)	−0.4338 (3)	0.4076 (3)	0.0302 (10)	
H22	0.2656	−0.4785	0.4440	0.036*	
C23	0.2451 (2)	−0.3228 (3)	0.4050 (2)	0.0250 (9)	
H23	0.2847	−0.2924	0.4393	0.030*	
C24	0.1652 (3)	−0.6017 (3)	0.3607 (3)	0.0542 (14)	
H24A	0.2076	−0.6399	0.3529	0.081*	
H24B	0.1284	−0.6225	0.3137	0.081*	
H24C	0.1523	−0.6216	0.4174	0.081*	
C25	0.2687 (2)	−0.1023 (3)	0.1512 (2)	0.0170 (9)	
C26	0.2772 (2)	−0.2220 (3)	0.1571 (2)	0.0174 (8)	
C27	0.2314 (2)	−0.2910 (3)	0.1080 (2)	0.0236 (9)	
H27	0.1943	−0.2613	0.0686	0.028*	
C28	0.2385 (2)	−0.4027 (3)	0.1151 (3)	0.0275 (10)	
H28	0.2058	−0.4485	0.0812	0.033*	
C29	0.2919 (2)	−0.4482 (3)	0.1703 (3)	0.0307 (10)	
C30	0.3389 (2)	−0.3788 (3)	0.2193 (2)	0.0323 (11)	
H30	0.3765	−0.4089	0.2575	0.039*	
C31	0.3318 (2)	−0.2670 (3)	0.2132 (2)	0.0263 (10)	
H31	0.3642	−0.2210	0.2474	0.032*	
C32	0.2998 (3)	−0.5691 (3)	0.1791 (3)	0.0514 (13)	
H32A	0.3443	−0.5909	0.1638	0.077*	
H32B	0.2629	−0.6050	0.1396	0.077*	
H32C	0.2973	−0.5905	0.2396	0.077*	
C33	0.66019 (19)	1.1070 (3)	0.1573 (2)	0.0124 (7)	
C34	0.60372 (19)	1.1704 (3)	0.1063 (2)	0.0140 (7)	
C35	0.54184 (19)	1.1833 (3)	0.1390 (2)	0.0162 (8)	
H35	0.5368	1.1533	0.1944	0.019*	
C36	0.4880 (2)	1.2394 (3)	0.0912 (2)	0.0207 (8)	
H36	0.4466	1.2489	0.1147	0.025*	
C37	0.4934 (2)	1.2820 (3)	0.0092 (2)	0.0202 (8)	
C38	0.5552 (2)	1.2687 (3)	−0.0227 (2)	0.0233 (9)	
H38	0.5602	1.2974	−0.0785	0.028*	
C39	0.6092 (2)	1.2145 (3)	0.0256 (2)	0.0178 (8)	
H39	0.6511	1.2072	0.0028	0.021*	
C40	0.4339 (2)	1.3391 (4)	−0.0450 (3)	0.0319 (10)	
H40A	0.4512	1.3983	−0.0781	0.048*	
H40B	0.4034	1.3690	−0.0064	0.048*	
H40C	0.4084	1.2872	−0.0858	0.048*	
C41	0.82912 (19)	1.1045 (3)	0.3500 (2)	0.0136 (7)	
C42	0.88448 (19)	1.1675 (3)	0.4058 (2)	0.0130 (7)	
C43	0.87193 (19)	1.2131 (3)	0.4849 (2)	0.0161 (8)	
H43	0.8283	1.2054	0.5034	0.019*	
C44	0.9243 (2)	1.2698 (3)	0.5357 (2)	0.0208 (8)	
H44	0.9157	1.3021	0.5888	0.025*	
C45	0.9888 (2)	1.2809 (3)	0.5114 (2)	0.0212 (9)	
C46	1.0001 (2)	1.2349 (3)	0.4332 (2)	0.0215 (8)	
H46	1.0440	1.2418	0.4153	0.026*	
C47	0.94859 (19)	1.1793 (3)	0.3805 (2)	0.0173 (8)	
H47	0.9572	1.1489	0.3267	0.021*	
C48	1.0455 (2)	1.3396 (3)	0.5700 (2)	0.0323 (10)	
H48A	1.0740	1.3795	0.5339	0.048*	
H48B	1.0739	1.2867	0.6065	0.048*	
H48C	1.0254	1.3910	0.6075	0.048*	
C49	0.6605 (2)	0.7893 (3)	0.3108 (2)	0.0151 (8)	
C50	0.6655 (2)	0.6689 (3)	0.3203 (2)	0.0176 (8)	
C51	0.6159 (2)	0.6022 (3)	0.2732 (2)	0.0171 (8)	
H51	0.5788	0.6337	0.2349	0.021*	
C52	0.6203 (2)	0.4904 (3)	0.2821 (2)	0.0229 (9)	
H52	0.5870	0.4459	0.2479	0.027*	
C53	0.6722 (2)	0.4418 (3)	0.3395 (2)	0.0246 (9)	
C54	0.7219 (2)	0.5089 (3)	0.3868 (2)	0.0241 (9)	
H54	0.7582	0.4776	0.4264	0.029*	
C55	0.7185 (2)	0.6202 (3)	0.3764 (2)	0.0202 (8)	
H55	0.7532	0.6645	0.4082	0.024*	
C56	0.6738 (2)	0.3198 (3)	0.3533 (3)	0.0362 (11)	
H56A	0.7216	0.2948	0.3643	0.054*	
H56B	0.6498	0.2837	0.3009	0.054*	
H56C	0.6512	0.3017	0.4038	0.054*	
C57	0.8255 (2)	0.8002 (3)	0.1982 (2)	0.0139 (8)	
C58	0.81646 (19)	0.6797 (3)	0.1965 (2)	0.0146 (8)	
C59	0.76113 (19)	0.6313 (3)	0.1440 (2)	0.0191 (8)	
H59	0.7280	0.6756	0.1094	0.023*	
C60	0.7538 (2)	0.5190 (3)	0.1416 (3)	0.0241 (9)	
H60	0.7158	0.4870	0.1052	0.029*	
C61	0.8017 (2)	0.4529 (3)	0.1921 (2)	0.0245 (9)	
C62	0.8570 (2)	0.5018 (3)	0.2451 (2)	0.0231 (9)	
H62	0.8897	0.4579	0.2808	0.028*	
C63	0.86452 (19)	0.6136 (3)	0.2460 (2)	0.0188 (8)	
H63	0.9032	0.6456	0.2810	0.023*	
C64	0.7929 (3)	0.3304 (3)	0.1886 (3)	0.0432 (12)	
H64A	0.8312	0.2960	0.2266	0.065*	
H64B	0.7921	0.3052	0.1283	0.065*	
H64C	0.7496	0.3108	0.2087	0.065*	
C65	0.5648 (2)	1.0193 (3)	0.4140 (2)	0.0149 (8)	
H65	0.5454	0.9784	0.3641	0.018*	
C66	0.52289 (19)	1.0508 (3)	0.4743 (2)	0.0126 (7)	
C67	0.5516 (2)	1.1111 (3)	0.5462 (2)	0.0160 (8)	
H67	0.5239	1.1348	0.5878	0.019*	
C68	0.6204 (2)	1.1368 (3)	0.5574 (2)	0.0197 (8)	
H68	0.6410	1.1775	0.6068	0.024*	
C69	0.6588 (2)	1.1020 (3)	0.4948 (2)	0.0161 (8)	
H69	0.7064	1.1191	0.5023	0.019*	
C70	0.4496 (2)	1.0156 (3)	0.4684 (2)	0.0148 (8)	
C71	0.9239 (2)	1.0286 (3)	0.0847 (2)	0.0161 (8)	
H71	0.9439	0.9865	0.1335	0.019*	
C72	0.96465 (19)	1.0578 (3)	0.0227 (2)	0.0163 (8)	
C73	0.9348 (2)	1.1208 (3)	−0.0482 (2)	0.0200 (9)	
H73	0.9611	1.1436	−0.0917	0.024*	
C74	0.8664 (2)	1.1497 (3)	−0.0544 (2)	0.0183 (8)	
H74	0.8450	1.1917	−0.1025	0.022*	
C75	0.8297 (2)	1.1165 (3)	0.0103 (2)	0.0160 (8)	
H75	0.7828	1.1368	0.0058	0.019*	
C76	1.0381 (2)	1.0218 (3)	0.0246 (2)	0.0177 (8)	

### Atomic displacement parameters (Å^2^)

**Table:**

	*U*^11^	*U*^22^	*U*^33^	*U*^12^	*U*^13^	*U*^23^
Zn1	0.0127 (2)	0.0163 (2)	0.01528 (19)	0.00210 (19)	0.00272 (16)	−0.00207 (17)
Zn2	0.0139 (2)	0.0175 (2)	0.0189 (2)	−0.00305 (19)	0.00077 (16)	0.00476 (18)
Zn3	0.0115 (3)	0.01344 (19)	0.01577 (19)	−0.00085 (17)	0.00219 (16)	0.00244 (17)
Zn4	0.0120 (3)	0.0133 (2)	0.0149 (2)	0.00080 (18)	0.00251 (16)	−0.00128 (16)
N1	0.0163 (19)	0.0153 (15)	0.0151 (13)	−0.0018 (13)	0.0031 (12)	0.0040 (12)
N2	0.012 (2)	0.0215 (17)	0.0204 (14)	−0.0003 (14)	0.0068 (13)	0.0002 (13)
N3	0.0102 (18)	0.0145 (15)	0.0171 (14)	−0.0025 (13)	0.0047 (11)	−0.0034 (12)
N4	0.0061 (19)	0.0269 (18)	0.0279 (16)	0.0040 (14)	0.0042 (13)	−0.0005 (14)
O1	0.0243 (17)	0.0180 (13)	0.0177 (12)	−0.0008 (12)	0.0018 (10)	−0.0010 (10)
O2	0.0142 (15)	0.0221 (14)	0.0253 (13)	−0.0050 (12)	0.0032 (10)	0.0042 (11)
O3	0.0140 (15)	0.0247 (14)	0.0213 (12)	0.0057 (12)	0.0033 (10)	0.0001 (10)
O4	0.0184 (16)	0.0249 (15)	0.0215 (13)	0.0009 (12)	−0.0006 (10)	0.0059 (11)
O5	0.0230 (17)	0.0218 (14)	0.0163 (11)	−0.0077 (12)	0.0016 (11)	0.0003 (10)
O6	0.0193 (16)	0.0210 (14)	0.0204 (12)	−0.0018 (12)	0.0008 (10)	0.0007 (11)
O7	0.0189 (16)	0.0178 (13)	0.0214 (13)	0.0065 (11)	0.0051 (11)	0.0021 (10)
O8	0.0264 (17)	0.0160 (13)	0.0210 (12)	0.0024 (12)	0.0000 (11)	−0.0016 (11)
O9	0.0156 (16)	0.0209 (13)	0.0163 (11)	0.0037 (11)	0.0033 (10)	−0.0003 (10)
O10	0.0137 (16)	0.0222 (14)	0.0217 (12)	−0.0038 (11)	0.0004 (10)	0.0036 (10)
O11	0.0159 (16)	0.0241 (14)	0.0185 (12)	0.0018 (12)	0.0031 (10)	0.0075 (11)
O12	0.0162 (17)	0.0170 (13)	0.0191 (12)	0.0023 (11)	0.0036 (10)	0.0002 (10)
O13	0.0103 (16)	0.0146 (13)	0.0237 (13)	−0.0032 (11)	0.0030 (10)	0.0002 (10)
O14	0.0154 (16)	0.0228 (14)	0.0166 (12)	−0.0010 (11)	0.0036 (10)	−0.0056 (10)
O15	0.0168 (17)	0.0206 (14)	0.0230 (13)	0.0020 (12)	0.0008 (11)	0.0020 (11)
O16	0.0161 (16)	0.0147 (12)	0.0199 (13)	−0.0024 (11)	0.0018 (11)	0.0014 (10)
O17	0.0160 (16)	0.0123 (12)	0.0191 (12)	0.0025 (11)	0.0009 (10)	−0.0006 (10)
O18	0.0136 (16)	0.0205 (14)	0.0269 (13)	−0.0023 (11)	0.0020 (11)	−0.0033 (11)
O19	0.0146 (16)	0.0226 (14)	0.0202 (12)	−0.0015 (12)	0.0081 (11)	0.0001 (10)
O20	0.0174 (17)	0.0257 (15)	0.0286 (14)	0.0040 (13)	0.0093 (12)	−0.0008 (12)
O21	0.015 (2)	0.073 (3)	0.0232 (15)	0.0114 (16)	−0.0023 (13)	−0.0068 (14)
O22	0.025 (2)	0.112 (3)	0.0313 (17)	−0.020 (2)	−0.0062 (15)	0.0273 (19)
C1	0.017 (2)	0.0107 (17)	0.0203 (17)	0.0053 (15)	0.0001 (15)	0.0024 (13)
C2	0.014 (2)	0.0141 (17)	0.0171 (16)	0.0005 (15)	0.0013 (14)	0.0037 (13)
C3	0.021 (2)	0.020 (2)	0.0217 (17)	−0.0030 (17)	0.0052 (15)	−0.0015 (15)
C4	0.029 (3)	0.029 (2)	0.0189 (18)	0.0005 (19)	−0.0038 (16)	−0.0077 (16)
C5	0.034 (3)	0.019 (2)	0.0249 (19)	−0.0094 (19)	−0.0104 (17)	−0.0005 (15)
C6	0.019 (2)	0.035 (2)	0.031 (2)	−0.0128 (19)	0.0011 (16)	0.0060 (17)
C7	0.024 (2)	0.031 (2)	0.0171 (17)	−0.0038 (18)	0.0023 (15)	0.0034 (16)
C8	0.048 (3)	0.028 (2)	0.043 (2)	−0.017 (2)	−0.021 (2)	0.001 (2)
C9	0.017 (2)	0.0074 (16)	0.0175 (16)	0.0003 (14)	−0.0015 (14)	−0.0015 (13)
C10	0.016 (2)	0.0117 (17)	0.0187 (16)	0.0014 (15)	−0.0005 (14)	−0.0005 (13)
C11	0.018 (2)	0.026 (2)	0.0171 (16)	0.0004 (17)	0.0024 (14)	−0.0006 (14)
C12	0.016 (2)	0.029 (2)	0.0292 (19)	0.0064 (17)	0.0050 (15)	−0.0073 (17)
C13	0.019 (2)	0.0136 (18)	0.0269 (18)	0.0034 (16)	−0.0042 (15)	−0.0029 (15)
C14	0.024 (3)	0.029 (2)	0.0245 (19)	0.0009 (18)	−0.0011 (16)	0.0057 (16)
C15	0.015 (2)	0.023 (2)	0.0194 (17)	0.0008 (16)	0.0047 (14)	0.0004 (15)
C16	0.026 (2)	0.027 (2)	0.042 (2)	0.0132 (19)	−0.0059 (18)	0.0008 (19)
C17	0.020 (2)	0.0186 (18)	0.0117 (15)	0.0003 (17)	0.0065 (14)	0.0001 (14)
C18	0.022 (2)	0.0156 (18)	0.0205 (17)	−0.0034 (17)	0.0090 (15)	−0.0028 (14)
C19	0.033 (3)	0.024 (2)	0.0156 (17)	−0.0065 (19)	−0.0038 (15)	0.0035 (15)
C20	0.051 (3)	0.028 (2)	0.025 (2)	−0.018 (2)	−0.0045 (19)	−0.0018 (17)
C21	0.052 (3)	0.022 (2)	0.0259 (19)	−0.011 (2)	0.0125 (19)	−0.0063 (16)
C22	0.033 (3)	0.017 (2)	0.041 (2)	0.0033 (19)	0.0071 (19)	0.0034 (17)
C23	0.020 (2)	0.019 (2)	0.035 (2)	−0.0002 (17)	0.0019 (16)	−0.0024 (16)
C24	0.087 (4)	0.018 (2)	0.059 (3)	−0.015 (3)	0.015 (3)	−0.004 (2)
C25	0.021 (2)	0.0171 (18)	0.0164 (16)	0.0038 (16)	0.0142 (16)	0.0041 (15)
C26	0.020 (2)	0.0185 (18)	0.0143 (15)	−0.0010 (17)	0.0058 (14)	0.0014 (15)
C27	0.014 (2)	0.020 (2)	0.035 (2)	−0.0005 (17)	−0.0005 (16)	0.0016 (16)
C28	0.028 (3)	0.020 (2)	0.033 (2)	−0.0034 (18)	0.0026 (17)	0.0013 (16)
C29	0.046 (3)	0.0159 (19)	0.031 (2)	0.002 (2)	0.0064 (19)	0.0046 (17)
C30	0.043 (3)	0.023 (2)	0.027 (2)	0.007 (2)	−0.0082 (19)	0.0052 (17)
C31	0.039 (3)	0.021 (2)	0.0172 (17)	0.0027 (19)	−0.0026 (16)	−0.0014 (15)
C32	0.063 (4)	0.028 (2)	0.057 (3)	0.007 (3)	−0.008 (3)	0.001 (2)
C33	0.013 (2)	0.0059 (15)	0.0176 (16)	−0.0033 (14)	0.0008 (14)	−0.0026 (13)
C34	0.015 (2)	0.0092 (16)	0.0166 (16)	−0.0023 (15)	−0.0010 (13)	−0.0031 (13)
C35	0.015 (2)	0.0168 (18)	0.0176 (16)	−0.0006 (15)	0.0036 (14)	0.0006 (14)
C36	0.015 (2)	0.023 (2)	0.0254 (18)	−0.0005 (17)	0.0054 (15)	0.0001 (15)
C37	0.015 (2)	0.0164 (18)	0.0268 (19)	0.0003 (16)	−0.0045 (15)	0.0016 (15)
C38	0.027 (3)	0.024 (2)	0.0186 (17)	0.0037 (18)	0.0027 (16)	0.0077 (15)
C39	0.015 (2)	0.0213 (19)	0.0181 (16)	0.0036 (16)	0.0066 (14)	0.0009 (15)
C40	0.022 (3)	0.035 (2)	0.036 (2)	0.009 (2)	−0.0044 (18)	0.0085 (19)
C41	0.013 (2)	0.0072 (15)	0.0200 (16)	0.0009 (15)	0.0019 (14)	0.0006 (13)
C42	0.014 (2)	0.0097 (16)	0.0146 (15)	−0.0008 (14)	0.0009 (13)	0.0021 (13)
C43	0.010 (2)	0.021 (2)	0.0180 (17)	−0.0031 (16)	0.0058 (14)	0.0003 (15)
C44	0.023 (2)	0.022 (2)	0.0175 (17)	−0.0028 (17)	0.0023 (15)	−0.0026 (15)
C45	0.022 (2)	0.0175 (19)	0.0225 (18)	−0.0026 (17)	−0.0031 (15)	−0.0013 (15)
C46	0.011 (2)	0.024 (2)	0.0301 (19)	−0.0044 (16)	0.0026 (15)	−0.0024 (16)
C47	0.019 (2)	0.0198 (19)	0.0140 (15)	−0.0004 (16)	0.0042 (14)	−0.0014 (14)
C48	0.024 (3)	0.034 (2)	0.036 (2)	−0.0047 (19)	−0.0053 (18)	−0.0100 (18)
C49	0.015 (2)	0.0191 (19)	0.0133 (15)	−0.0023 (16)	0.0075 (14)	0.0030 (14)
C50	0.022 (2)	0.0149 (18)	0.0174 (16)	0.0022 (16)	0.0096 (15)	0.0029 (14)
C51	0.018 (2)	0.0178 (18)	0.0159 (16)	−0.0023 (16)	0.0045 (14)	0.0022 (14)
C52	0.032 (3)	0.021 (2)	0.0169 (17)	−0.0047 (18)	0.0072 (16)	−0.0020 (15)
C53	0.031 (3)	0.0122 (19)	0.033 (2)	0.0025 (17)	0.0135 (18)	0.0037 (16)
C54	0.022 (2)	0.020 (2)	0.030 (2)	0.0038 (17)	0.0038 (16)	0.0064 (16)
C55	0.013 (2)	0.021 (2)	0.0259 (18)	0.0013 (17)	0.0020 (15)	0.0020 (15)
C56	0.046 (3)	0.013 (2)	0.050 (3)	0.002 (2)	0.009 (2)	0.0016 (18)
C57	0.017 (2)	0.0149 (17)	0.0113 (15)	0.0013 (16)	0.0071 (14)	0.0012 (13)
C58	0.016 (2)	0.0145 (18)	0.0141 (15)	0.0007 (15)	0.0041 (13)	0.0005 (14)
C59	0.017 (2)	0.0152 (19)	0.0254 (18)	0.0017 (16)	0.0028 (15)	−0.0028 (15)
C60	0.020 (2)	0.021 (2)	0.032 (2)	−0.0043 (17)	0.0073 (17)	−0.0032 (16)
C61	0.033 (2)	0.0109 (18)	0.033 (2)	0.0032 (17)	0.0156 (18)	0.0018 (15)
C62	0.029 (2)	0.0191 (19)	0.0217 (18)	0.0044 (17)	0.0050 (16)	0.0018 (15)
C63	0.017 (2)	0.0184 (19)	0.0218 (17)	0.0012 (16)	0.0059 (15)	−0.0007 (14)
C64	0.051 (3)	0.017 (2)	0.066 (3)	0.000 (2)	0.021 (3)	0.001 (2)
C65	0.013 (2)	0.0140 (18)	0.0173 (16)	−0.0012 (15)	0.0001 (14)	0.0014 (14)
C66	0.010 (2)	0.0111 (17)	0.0162 (15)	0.0028 (14)	0.0017 (13)	0.0056 (13)
C67	0.014 (2)	0.0174 (18)	0.0179 (17)	−0.0014 (16)	0.0051 (14)	0.0015 (14)
C68	0.025 (3)	0.0165 (18)	0.0174 (17)	−0.0045 (17)	0.0032 (15)	−0.0013 (14)
C69	0.015 (2)	0.0169 (18)	0.0162 (16)	−0.0021 (16)	0.0003 (14)	0.0078 (14)
C70	0.013 (2)	0.0107 (17)	0.0221 (18)	0.0026 (15)	0.0057 (15)	0.0019 (14)
C71	0.014 (2)	0.0142 (17)	0.0195 (17)	0.0022 (16)	0.0015 (15)	−0.0008 (14)
C72	0.014 (2)	0.0163 (18)	0.0188 (17)	−0.0014 (16)	0.0039 (14)	−0.0053 (14)
C73	0.031 (3)	0.0155 (19)	0.0145 (17)	−0.0009 (17)	0.0082 (16)	−0.0008 (14)
C74	0.018 (2)	0.0193 (19)	0.0164 (16)	−0.0002 (16)	0.0001 (14)	−0.0006 (14)
C75	0.012 (2)	0.0176 (18)	0.0180 (16)	0.0007 (15)	0.0000 (14)	−0.0027 (14)
C76	0.019 (2)	0.0115 (18)	0.0228 (18)	−0.0029 (16)	0.0039 (16)	−0.0032 (14)

### Geometric parameters (Å, °)

**Table:**

Zn1—O1	1.964 (2)	C24—H24A	0.9800
Zn1—O3	1.939 (3)	C24—H24B	0.9800
Zn1—O7	2.505 (3)	C24—H24C	0.9800
Zn1—O8	1.975 (2)	C25—C26	1.482 (5)
Zn1—O9	1.966 (2)	C26—C27	1.379 (5)
Zn2—O2	1.942 (3)	C26—C31	1.390 (5)
Zn2—O4	1.967 (2)	C27—C28	1.382 (5)
Zn2—O5	2.467 (3)	C27—H27	0.9500
Zn2—O6	1.989 (3)	C28—C29	1.370 (6)
Zn2—O10	1.994 (2)	C28—H28	0.9500
Zn3—O11	1.926 (2)	C29—C30	1.394 (6)
Zn3—O13	1.967 (3)	C29—C32	1.498 (5)
Zn3—O16	1.932 (2)	C30—C31	1.383 (5)
Zn3—N1	2.047 (3)	C30—H30	0.9500
Zn4—O12	1.968 (3)	C31—H31	0.9500
Zn4—O14	1.924 (2)	C32—H32A	0.9800
Zn4—O17	1.940 (2)	C32—H32B	0.9800
Zn4—N3	2.036 (3)	C32—H32C	0.9800
N1—C65	1.336 (5)	C33—C34	1.484 (5)
N1—C69	1.347 (4)	C34—C39	1.378 (4)
N2—C70	1.328 (4)	C34—C35	1.398 (5)
N2—H2A	0.8800	C35—C36	1.382 (5)
N2—H2B	0.8800	C35—H35	0.9500
N3—C71	1.339 (5)	C36—C37	1.389 (5)
N3—C75	1.341 (4)	C36—H36	0.9500
N4—C76	1.318 (5)	C37—C38	1.391 (5)
N4—H4A	0.8800	C37—C40	1.507 (5)
N4—H4B	0.8800	C38—C39	1.375 (5)
O1—C1	1.255 (4)	C38—H38	0.9500
O2—C1	1.267 (4)	C39—H39	0.9500
O3—C9	1.255 (4)	C40—H40A	0.9800
O4—C9	1.261 (4)	C40—H40B	0.9800
O5—C17	1.262 (4)	C40—H40C	0.9800
O6—C17	1.275 (4)	C41—C42	1.500 (5)
O7—C25	1.268 (4)	C42—C47	1.385 (5)
O8—C25	1.267 (5)	C42—C43	1.399 (4)
O9—H91	0.9738	C43—C44	1.386 (5)
O9—H92	0.9734	C43—H43	0.9500
O10—H101	0.9719	C44—C45	1.385 (5)
O10—H102	0.9734	C44—H44	0.9500
O11—C33	1.258 (4)	C45—C46	1.383 (5)
O12—C33	1.272 (4)	C45—C48	1.510 (5)
O13—C41	1.261 (4)	C46—C47	1.382 (5)
O14—C41	1.256 (4)	C46—H46	0.9500
O15—C49	1.247 (4)	C47—H47	0.9500
O16—C49	1.290 (4)	C48—H48A	0.9800
O17—C57	1.295 (4)	C48—H48B	0.9800
O18—C57	1.233 (4)	C48—H48C	0.9800
O19—C70	1.257 (4)	C49—C50	1.489 (5)
O20—C76	1.235 (4)	C50—C55	1.388 (5)
O21—H211	0.9712	C50—C51	1.393 (5)
O21—H212	0.8577	C51—C52	1.382 (5)
O22—H221	0.9708	C51—H51	0.9500
O22—H222	0.9737	C52—C53	1.384 (5)
C1—C2	1.496 (5)	C52—H52	0.9500
C2—C7	1.386 (5)	C53—C54	1.399 (5)
C2—C3	1.387 (4)	C53—C56	1.514 (5)
C3—C4	1.391 (5)	C54—C55	1.377 (5)
C3—H3	0.9500	C54—H54	0.9500
C4—C5	1.381 (5)	C55—H55	0.9500
C4—H4	0.9500	C56—H56A	0.9800
C5—C6	1.383 (5)	C56—H56B	0.9800
C5—C8	1.522 (5)	C56—H56C	0.9800
C6—C7	1.383 (5)	C57—C58	1.490 (5)
C6—H6	0.9500	C58—C63	1.387 (5)
C7—H7	0.9500	C58—C59	1.390 (5)
C8—H8A	0.9800	C59—C60	1.388 (5)
C8—H8B	0.9800	C59—H59	0.9500
C8—H8C	0.9800	C60—C61	1.391 (5)
C9—C10	1.485 (5)	C60—H60	0.9500
C10—C15	1.390 (5)	C61—C62	1.396 (6)
C10—C11	1.398 (5)	C61—C64	1.516 (5)
C11—C12	1.380 (5)	C62—C63	1.381 (5)
C11—H11	0.9500	C62—H62	0.9500
C12—C13	1.391 (5)	C63—H63	0.9500
C12—H12	0.9500	C64—H64A	0.9800
C13—C14	1.386 (5)	C64—H64B	0.9800
C13—C16	1.501 (5)	C64—H64C	0.9800
C14—C15	1.391 (5)	C65—C66	1.391 (5)
C14—H14	0.9500	C65—H65	0.9500
C15—H15	0.9500	C66—C67	1.383 (5)
C16—H16A	0.9800	C66—C70	1.496 (5)
C16—H16B	0.9800	C67—C68	1.378 (5)
C16—H16C	0.9800	C67—H67	0.9500
C17—C18	1.486 (5)	C68—C69	1.382 (5)
C18—C19	1.385 (5)	C68—H68	0.9500
C18—C23	1.392 (5)	C69—H69	0.9500
C19—C20	1.377 (5)	C71—C72	1.387 (5)
C19—H19	0.9500	C71—H71	0.9500
C20—C21	1.384 (6)	C72—C73	1.397 (5)
C20—H20	0.9500	C72—C76	1.509 (5)
C21—C22	1.387 (6)	C73—C74	1.382 (5)
C21—C24	1.507 (5)	C73—H73	0.9500
C22—C23	1.384 (5)	C74—C75	1.381 (5)
C22—H22	0.9500	C74—H74	0.9500
C23—H23	0.9500	C75—H75	0.9500
			
O3—Zn1—O1	107.92 (10)	C26—C27—H27	119.5
O3—Zn1—O9	101.01 (10)	C28—C27—H27	119.5
O1—Zn1—O9	102.14 (10)	C29—C28—C27	120.9 (4)
O3—Zn1—O8	135.45 (11)	C29—C28—H28	119.5
O1—Zn1—O8	97.87 (10)	C27—C28—H28	119.5
O9—Zn1—O8	108.41 (10)	C28—C29—C30	118.2 (4)
O3—Zn1—C25	114.78 (12)	C28—C29—C32	121.4 (4)
O1—Zn1—C25	126.49 (11)	C30—C29—C32	120.4 (4)
O9—Zn1—C25	99.84 (10)	C31—C30—C29	121.2 (4)
O8—Zn1—C25	28.64 (11)	C31—C30—H30	119.4
O2—Zn2—O4	107.30 (10)	C29—C30—H30	119.4
O2—Zn2—O6	142.57 (11)	C30—C31—C26	119.9 (4)
O4—Zn2—O6	100.23 (11)	C30—C31—H31	120.1
O2—Zn2—O10	97.18 (10)	C26—C31—H31	120.1
O4—Zn2—O10	99.04 (10)	C29—C32—H32A	109.5
O6—Zn2—O10	103.10 (10)	C29—C32—H32B	109.5
O2—Zn2—O5	90.36 (10)	H32A—C32—H32B	109.5
O4—Zn2—O5	157.49 (9)	C29—C32—H32C	109.5
O6—Zn2—O5	58.03 (9)	H32A—C32—H32C	109.5
O10—Zn2—O5	92.28 (9)	H32B—C32—H32C	109.5
O2—Zn2—C17	117.82 (12)	O11—C33—O12	124.9 (3)
O4—Zn2—C17	129.36 (11)	O11—C33—C34	116.8 (3)
O6—Zn2—C17	29.17 (11)	O12—C33—C34	118.3 (3)
O10—Zn2—C17	97.06 (10)	C39—C34—C35	118.4 (3)
O5—Zn2—C17	28.95 (10)	C39—C34—C33	122.1 (3)
O11—Zn3—O16	126.93 (10)	C35—C34—C33	119.5 (3)
O11—Zn3—O13	107.64 (10)	C36—C35—C34	120.2 (3)
O16—Zn3—O13	109.04 (11)	C36—C35—H35	119.9
O11—Zn3—N1	101.55 (11)	C34—C35—H35	119.9
O16—Zn3—N1	109.37 (11)	C35—C36—C37	121.2 (4)
O13—Zn3—N1	98.49 (11)	C35—C36—H36	119.4
O14—Zn4—O17	124.32 (10)	C37—C36—H36	119.4
O14—Zn4—O12	107.99 (10)	C36—C37—C38	118.0 (3)
O17—Zn4—O12	107.01 (11)	C36—C37—C40	121.5 (4)
O14—Zn4—N3	103.33 (11)	C38—C37—C40	120.5 (3)
O17—Zn4—N3	112.65 (11)	C39—C38—C37	120.8 (3)
O12—Zn4—N3	98.50 (11)	C39—C38—H38	119.6
C65—N1—C69	118.6 (3)	C37—C38—H38	119.6
C65—N1—Zn3	122.1 (2)	C38—C39—C34	121.4 (4)
C69—N1—Zn3	119.3 (3)	C38—C39—H39	119.3
C70—N2—H2A	120.0	C34—C39—H39	119.3
C70—N2—H2B	120.0	C37—C40—H40A	109.5
H2A—N2—H2B	120.0	C37—C40—H40B	109.5
C71—N3—C75	118.2 (3)	H40A—C40—H40B	109.5
C71—N3—Zn4	122.6 (2)	C37—C40—H40C	109.5
C75—N3—Zn4	119.2 (2)	H40A—C40—H40C	109.5
C76—N4—H4A	120.0	H40B—C40—H40C	109.5
C76—N4—H4B	120.0	O14—C41—O13	125.7 (3)
H4A—N4—H4B	120.0	O14—C41—C42	116.3 (3)
C1—O1—Zn1	127.3 (2)	O13—C41—C42	118.1 (3)
C1—O2—Zn2	137.6 (2)	C47—C42—C43	119.6 (3)
C9—O3—Zn1	127.7 (2)	C47—C42—C41	120.4 (3)
C9—O4—Zn2	137.8 (3)	C43—C42—C41	120.0 (3)
C17—O5—Zn2	79.8 (2)	C44—C43—C42	118.7 (3)
C17—O6—Zn2	101.3 (2)	C44—C43—H43	120.6
C25—O8—Zn1	103.0 (2)	C42—C43—H43	120.6
Zn1—O9—H91	116.3	C45—C44—C43	122.1 (3)
Zn1—O9—H92	109.9	C45—C44—H44	119.0
H91—O9—H92	106.5	C43—C44—H44	119.0
Zn2—O10—H101	113.1	C46—C45—C44	118.2 (3)
Zn2—O10—H102	103.6	C46—C45—C48	121.1 (4)
H101—O10—H102	106.5	C44—C45—C48	120.7 (3)
C33—O11—Zn3	143.6 (3)	C47—C46—C45	121.0 (4)
C33—O12—Zn4	130.1 (2)	C47—C46—H46	119.5
C41—O13—Zn3	131.9 (2)	C45—C46—H46	119.5
C41—O14—Zn4	141.2 (3)	C46—C47—C42	120.4 (3)
C49—O16—Zn3	109.4 (2)	C46—C47—H47	119.8
C57—O17—Zn4	108.2 (2)	C42—C47—H47	119.8
H211—O21—H212	110.9	C45—C48—H48A	109.5
H221—O22—H222	106.5	C45—C48—H48B	109.5
O1—C1—O2	125.4 (3)	H48A—C48—H48B	109.5
O1—C1—C2	117.3 (3)	C45—C48—H48C	109.5
O2—C1—C2	117.3 (3)	H48A—C48—H48C	109.5
C7—C2—C3	119.1 (3)	H48B—C48—H48C	109.5
C7—C2—C1	119.8 (3)	O15—C49—O16	122.8 (3)
C3—C2—C1	121.0 (3)	O15—C49—C50	121.0 (3)
C2—C3—C4	119.9 (4)	O16—C49—C50	116.2 (3)
C2—C3—H3	120.0	C55—C50—C51	118.4 (3)
C4—C3—H3	120.0	C55—C50—C49	121.5 (3)
C5—C4—C3	120.9 (3)	C51—C50—C49	120.2 (3)
C5—C4—H4	119.5	C52—C51—C50	120.3 (4)
C3—C4—H4	119.5	C52—C51—H51	119.9
C4—C5—C6	118.8 (3)	C50—C51—H51	119.9
C4—C5—C8	121.4 (3)	C51—C52—C53	121.5 (4)
C6—C5—C8	119.7 (4)	C51—C52—H52	119.3
C5—C6—C7	120.7 (4)	C53—C52—H52	119.3
C5—C6—H6	119.6	C52—C53—C54	118.1 (3)
C7—C6—H6	119.6	C52—C53—C56	121.0 (4)
C6—C7—C2	120.5 (3)	C54—C53—C56	120.8 (4)
C6—C7—H7	119.8	C55—C54—C53	120.4 (4)
C2—C7—H7	119.8	C55—C54—H54	119.8
C5—C8—H8A	109.5	C53—C54—H54	119.8
C5—C8—H8B	109.5	C54—C55—C50	121.3 (4)
H8A—C8—H8B	109.5	C54—C55—H55	119.3
C5—C8—H8C	109.5	C50—C55—H55	119.3
H8A—C8—H8C	109.5	C53—C56—H56A	109.5
H8B—C8—H8C	109.5	C53—C56—H56B	109.5
O3—C9—O4	125.1 (3)	H56A—C56—H56B	109.5
O3—C9—C10	117.3 (3)	C53—C56—H56C	109.5
O4—C9—C10	117.7 (3)	H56A—C56—H56C	109.5
C15—C10—C11	117.9 (3)	H56B—C56—H56C	109.5
C15—C10—C9	121.3 (3)	O18—C57—O17	122.6 (3)
C11—C10—C9	120.8 (3)	O18—C57—C58	121.6 (3)
C12—C11—C10	121.1 (3)	O17—C57—C58	115.8 (3)
C12—C11—H11	119.4	C63—C58—C59	118.7 (3)
C10—C11—H11	119.4	C63—C58—C57	120.2 (3)
C11—C12—C13	121.1 (3)	C59—C58—C57	121.1 (3)
C11—C12—H12	119.5	C60—C59—C58	120.7 (3)
C13—C12—H12	119.5	C60—C59—H59	119.6
C14—C13—C12	117.9 (3)	C58—C59—H59	119.6
C14—C13—C16	120.9 (3)	C59—C60—C61	120.4 (4)
C12—C13—C16	121.2 (3)	C59—C60—H60	119.8
C13—C14—C15	121.4 (3)	C61—C60—H60	119.8
C13—C14—H14	119.3	C60—C61—C62	118.8 (3)
C15—C14—H14	119.3	C60—C61—C64	119.7 (4)
C10—C15—C14	120.6 (3)	C62—C61—C64	121.6 (4)
C10—C15—H15	119.7	C63—C62—C61	120.4 (4)
C14—C15—H15	119.7	C63—C62—H62	119.8
C13—C16—H16A	109.5	C61—C62—H62	119.8
C13—C16—H16B	109.5	C62—C63—C58	121.0 (4)
H16A—C16—H16B	109.5	C62—C63—H63	119.5
C13—C16—H16C	109.5	C58—C63—H63	119.5
H16A—C16—H16C	109.5	C61—C64—H64A	109.5
H16B—C16—H16C	109.5	C61—C64—H64B	109.5
O5—C17—O6	120.4 (3)	H64A—C64—H64B	109.5
O5—C17—C18	120.9 (3)	C61—C64—H64C	109.5
O6—C17—C18	118.6 (3)	H64A—C64—H64C	109.5
O5—C17—Zn2	71.2 (2)	H64B—C64—H64C	109.5
O6—C17—Zn2	49.49 (17)	N1—C65—C66	122.2 (3)
C18—C17—Zn2	166.5 (3)	N1—C65—H65	118.9
C19—C18—C23	118.0 (3)	C66—C65—H65	118.9
C19—C18—C17	121.6 (4)	C67—C66—C65	118.4 (3)
C23—C18—C17	120.4 (4)	C67—C66—C70	118.5 (3)
C20—C19—C18	120.9 (4)	C65—C66—C70	123.0 (3)
C20—C19—H19	119.6	C68—C67—C66	119.9 (3)
C18—C19—H19	119.6	C68—C67—H67	120.0
C19—C20—C21	121.5 (4)	C66—C67—H67	120.0
C19—C20—H20	119.3	C67—C68—C69	118.3 (3)
C21—C20—H20	119.3	C67—C68—H68	120.8
C20—C21—C22	117.9 (4)	C69—C68—H68	120.8
C20—C21—C24	121.7 (4)	N1—C69—C68	122.6 (4)
C22—C21—C24	120.4 (4)	N1—C69—H69	118.7
C23—C22—C21	120.9 (4)	C68—C69—H69	118.7
C23—C22—H22	119.5	O19—C70—N2	122.2 (4)
C21—C22—H22	119.5	O19—C70—C66	118.0 (3)
C22—C23—C18	120.8 (4)	N2—C70—C66	119.8 (3)
C22—C23—H23	119.6	N3—C71—C72	123.3 (3)
C18—C23—H23	119.6	N3—C71—H71	118.4
C21—C24—H24A	109.5	C72—C71—H71	118.4
C21—C24—H24B	109.5	C71—C72—C73	117.8 (4)
H24A—C24—H24B	109.5	C71—C72—C76	124.3 (3)
C21—C24—H24C	109.5	C73—C72—C76	117.9 (3)
H24A—C24—H24C	109.5	C74—C73—C72	119.1 (3)
H24B—C24—H24C	109.5	C74—C73—H73	120.4
O8—C25—O7	120.5 (3)	C72—C73—H73	120.4
O8—C25—C26	120.0 (3)	C75—C74—C73	119.1 (3)
O7—C25—C26	119.6 (4)	C75—C74—H74	120.4
O8—C25—Zn1	48.34 (17)	C73—C74—H74	120.4
O7—C25—Zn1	72.5 (2)	N3—C75—C74	122.5 (3)
C26—C25—Zn1	166.7 (3)	N3—C75—H75	118.7
C27—C26—C31	118.7 (3)	C74—C75—H75	118.7
C27—C26—C25	121.2 (4)	O20—C76—N4	123.7 (4)
C31—C26—C25	120.2 (4)	O20—C76—C72	117.8 (3)
C26—C27—C28	121.1 (4)	N4—C76—C72	118.5 (3)
			
O11—Zn3—N1—C65	61.7 (3)	C17—C18—C23—C22	−178.0 (3)
O16—Zn3—N1—C65	−74.5 (3)	Zn1—O8—C25—O7	−7.9 (4)
O13—Zn3—N1—C65	171.8 (3)	Zn1—O8—C25—C26	172.1 (3)
O11—Zn3—N1—C69	−118.8 (3)	O3—Zn1—C25—O8	−142.4 (2)
O16—Zn3—N1—C69	105.0 (3)	O1—Zn1—C25—O8	−2.6 (3)
O13—Zn3—N1—C69	−8.7 (3)	O9—Zn1—C25—O8	110.6 (2)
O14—Zn4—N3—C71	63.8 (3)	O3—Zn1—C25—O7	30.5 (2)
O17—Zn4—N3—C71	−72.9 (3)	O1—Zn1—C25—O7	170.20 (18)
O12—Zn4—N3—C71	174.6 (3)	O9—Zn1—C25—O7	−76.6 (2)
O14—Zn4—N3—C75	−117.9 (3)	O8—Zn1—C25—O7	172.8 (4)
O17—Zn4—N3—C75	105.4 (3)	O3—Zn1—C25—C26	−173.6 (11)
O12—Zn4—N3—C75	−7.1 (3)	O1—Zn1—C25—C26	−33.8 (11)
O3—Zn1—O1—C1	77.9 (3)	O9—Zn1—C25—C26	79.4 (11)
O9—Zn1—O1—C1	−176.1 (3)	O8—Zn1—C25—C26	−31.2 (10)
O8—Zn1—O1—C1	−65.3 (3)	O8—C25—C26—C27	171.7 (4)
C25—Zn1—O1—C1	−64.0 (3)	O7—C25—C26—C27	−8.3 (5)
O4—Zn2—O2—C1	−57.6 (4)	Zn1—C25—C26—C27	−161.8 (9)
O6—Zn2—O2—C1	77.8 (4)	O8—C25—C26—C31	−7.8 (5)
O10—Zn2—O2—C1	−159.5 (4)	O7—C25—C26—C31	172.2 (3)
O5—Zn2—O2—C1	108.2 (4)	Zn1—C25—C26—C31	18.7 (13)
C17—Zn2—O2—C1	98.6 (4)	C31—C26—C27—C28	1.1 (6)
O1—Zn1—O3—C9	−76.5 (3)	C25—C26—C27—C28	−178.4 (4)
O9—Zn1—O3—C9	176.7 (3)	C26—C27—C28—C29	−0.9 (6)
O8—Zn1—O3—C9	45.7 (3)	C27—C28—C29—C30	0.1 (6)
C25—Zn1—O3—C9	70.4 (3)	C27—C28—C29—C32	179.5 (4)
O2—Zn2—O4—C9	60.2 (4)	C28—C29—C30—C31	0.6 (7)
O6—Zn2—O4—C9	−94.1 (3)	C32—C29—C30—C31	−178.8 (4)
O10—Zn2—O4—C9	160.7 (3)	C29—C30—C31—C26	−0.4 (6)
O5—Zn2—O4—C9	−80.0 (5)	C27—C26—C31—C30	−0.4 (6)
C17—Zn2—O4—C9	−92.3 (4)	C25—C26—C31—C30	179.1 (4)
O2—Zn2—O5—C17	−162.3 (2)	Zn3—O11—C33—O12	7.3 (6)
O4—Zn2—O5—C17	−20.0 (4)	Zn3—O11—C33—C34	−173.4 (3)
O6—Zn2—O5—C17	−3.6 (2)	Zn4—O12—C33—O11	1.7 (5)
O10—Zn2—O5—C17	100.5 (2)	Zn4—O12—C33—C34	−177.6 (2)
O2—Zn2—O6—C17	40.1 (3)	O11—C33—C34—C39	179.5 (3)
O4—Zn2—O6—C17	177.2 (2)	O12—C33—C34—C39	−1.1 (5)
O10—Zn2—O6—C17	−80.9 (2)	O11—C33—C34—C35	−2.9 (5)
O5—Zn2—O6—C17	3.5 (2)	O12—C33—C34—C35	176.5 (3)
O3—Zn1—O8—C25	52.2 (3)	C39—C34—C35—C36	−0.3 (5)
O1—Zn1—O8—C25	177.9 (2)	C33—C34—C35—C36	−178.1 (3)
O9—Zn1—O8—C25	−76.5 (2)	C34—C35—C36—C37	1.4 (5)
O16—Zn3—O11—C33	−77.5 (4)	C35—C36—C37—C38	−1.4 (5)
O13—Zn3—O11—C33	54.4 (4)	C35—C36—C37—C40	177.4 (4)
N1—Zn3—O11—C33	157.3 (4)	C36—C37—C38—C39	0.3 (6)
O14—Zn4—O12—C33	−64.0 (3)	C40—C37—C38—C39	−178.5 (4)
O17—Zn4—O12—C33	72.0 (3)	C37—C38—C39—C34	0.8 (6)
N3—Zn4—O12—C33	−171.1 (3)	C35—C34—C39—C38	−0.8 (5)
O11—Zn3—O13—C41	−60.7 (3)	C33—C34—C39—C38	176.9 (3)
O16—Zn3—O13—C41	80.3 (3)	Zn4—O14—C41—O13	5.8 (6)
N1—Zn3—O13—C41	−165.7 (3)	Zn4—O14—C41—C42	−175.5 (3)
O17—Zn4—O14—C41	−68.0 (4)	Zn3—O13—C41—O14	0.6 (5)
O12—Zn4—O14—C41	58.4 (4)	Zn3—O13—C41—C42	−178.1 (2)
N3—Zn4—O14—C41	162.1 (4)	O14—C41—C42—C47	−10.0 (5)
O11—Zn3—O16—C49	−43.4 (3)	O13—C41—C42—C47	168.8 (3)
O13—Zn3—O16—C49	−174.8 (2)	O14—C41—C42—C43	171.2 (3)
N1—Zn3—O16—C49	78.6 (2)	O13—C41—C42—C43	−10.0 (5)
O14—Zn4—O17—C57	−45.3 (3)	C47—C42—C43—C44	0.4 (5)
O12—Zn4—O17—C57	−172.2 (2)	C41—C42—C43—C44	179.2 (3)
N3—Zn4—O17—C57	80.7 (2)	C42—C43—C44—C45	−1.2 (5)
Zn1—O1—C1—O2	−3.3 (5)	C43—C44—C45—C46	1.1 (5)
Zn1—O1—C1—C2	176.7 (2)	C43—C44—C45—C48	−177.7 (4)
Zn2—O2—C1—O1	−2.8 (6)	C44—C45—C46—C47	−0.2 (5)
Zn2—O2—C1—C2	177.1 (2)	C48—C45—C46—C47	178.7 (3)
O1—C1—C2—C7	−3.9 (5)	C45—C46—C47—C42	−0.6 (6)
O2—C1—C2—C7	176.1 (3)	C43—C42—C47—C46	0.5 (5)
O1—C1—C2—C3	175.4 (3)	C41—C42—C47—C46	−178.3 (3)
O2—C1—C2—C3	−4.5 (5)	Zn3—O16—C49—O15	2.5 (4)
C7—C2—C3—C4	−0.9 (5)	Zn3—O16—C49—C50	−176.1 (2)
C1—C2—C3—C4	179.7 (3)	O15—C49—C50—C55	−169.5 (3)
C2—C3—C4—C5	−0.3 (6)	O16—C49—C50—C55	9.1 (5)
C3—C4—C5—C6	1.1 (6)	O15—C49—C50—C51	9.7 (5)
C3—C4—C5—C8	−177.9 (4)	O16—C49—C50—C51	−171.8 (3)
C4—C5—C6—C7	−0.8 (6)	C55—C50—C51—C52	−0.7 (5)
C8—C5—C6—C7	178.3 (4)	C49—C50—C51—C52	−179.8 (3)
C5—C6—C7—C2	−0.5 (6)	C50—C51—C52—C53	2.3 (5)
C3—C2—C7—C6	1.3 (6)	C51—C52—C53—C54	−2.2 (5)
C1—C2—C7—C6	−179.4 (3)	C51—C52—C53—C56	175.7 (4)
Zn1—O3—C9—O4	3.2 (5)	C52—C53—C54—C55	0.5 (6)
Zn1—O3—C9—C10	−177.0 (2)	C56—C53—C54—C55	−177.5 (4)
Zn2—O4—C9—O3	0.7 (6)	C53—C54—C55—C50	1.2 (6)
Zn2—O4—C9—C10	−179.1 (2)	C51—C50—C55—C54	−1.0 (5)
O3—C9—C10—C15	1.7 (5)	C49—C50—C55—C54	178.1 (3)
O4—C9—C10—C15	−178.5 (3)	Zn4—O17—C57—O18	0.2 (4)
O3—C9—C10—C11	−179.6 (3)	Zn4—O17—C57—C58	−179.4 (2)
O4—C9—C10—C11	0.2 (5)	O18—C57—C58—C63	8.9 (5)
C15—C10—C11—C12	−0.4 (5)	O17—C57—C58—C63	−171.5 (3)
C9—C10—C11—C12	−179.1 (3)	O18—C57—C58—C59	−169.4 (3)
C10—C11—C12—C13	0.5 (6)	O17—C57—C58—C59	10.2 (5)
C11—C12—C13—C14	0.0 (6)	C63—C58—C59—C60	0.5 (5)
C11—C12—C13—C16	178.5 (3)	C57—C58—C59—C60	178.8 (3)
C12—C13—C14—C15	−0.5 (6)	C58—C59—C60—C61	0.3 (6)
C16—C13—C14—C15	−179.0 (4)	C59—C60—C61—C62	0.0 (6)
C11—C10—C15—C14	−0.1 (5)	C59—C60—C61—C64	−180.0 (4)
C9—C10—C15—C14	178.6 (3)	C60—C61—C62—C63	−1.1 (5)
C13—C14—C15—C10	0.6 (6)	C64—C61—C62—C63	178.9 (4)
Zn2—O5—C17—O6	5.5 (3)	C61—C62—C63—C58	1.9 (5)
Zn2—O5—C17—C18	−173.5 (3)	C59—C58—C63—C62	−1.6 (5)
Zn2—O6—C17—O5	−6.8 (4)	C57—C58—C63—C62	−179.9 (3)
Zn2—O6—C17—C18	172.2 (3)	C69—N1—C65—C66	0.4 (5)
O2—Zn2—C17—O5	20.1 (2)	Zn3—N1—C65—C66	179.9 (2)
O4—Zn2—C17—O5	170.26 (17)	N1—C65—C66—C67	0.6 (5)
O6—Zn2—C17—O5	173.8 (4)	N1—C65—C66—C70	−174.9 (3)
O10—Zn2—C17—O5	−81.9 (2)	C65—C66—C67—C68	−1.2 (5)
O2—Zn2—C17—O6	−153.7 (2)	C70—C66—C67—C68	174.5 (3)
O4—Zn2—C17—O6	−3.5 (3)	C66—C67—C68—C69	0.8 (5)
O10—Zn2—C17—O6	104.3 (2)	C65—N1—C69—C68	−0.9 (5)
O5—Zn2—C17—O6	−173.8 (4)	Zn3—N1—C69—C68	179.5 (3)
O2—Zn2—C17—C18	175.6 (10)	C67—C68—C69—N1	0.3 (5)
O4—Zn2—C17—C18	−34.2 (11)	C67—C66—C70—O19	−13.7 (5)
O6—Zn2—C17—C18	−30.7 (9)	C65—C66—C70—O19	161.8 (3)
O10—Zn2—C17—C18	73.6 (10)	C67—C66—C70—N2	166.0 (3)
O5—Zn2—C17—C18	155.5 (11)	C65—C66—C70—N2	−18.5 (5)
O5—C17—C18—C19	175.2 (3)	C75—N3—C71—C72	−0.2 (5)
O6—C17—C18—C19	−3.8 (5)	Zn4—N3—C71—C72	178.1 (3)
Zn2—C17—C18—C19	22.4 (12)	N3—C71—C72—C73	0.8 (5)
O5—C17—C18—C23	−6.8 (5)	N3—C71—C72—C76	−176.3 (3)
O6—C17—C18—C23	174.2 (3)	C71—C72—C73—C74	−1.1 (5)
Zn2—C17—C18—C23	−159.5 (8)	C76—C72—C73—C74	176.3 (3)
C23—C18—C19—C20	−0.3 (6)	C72—C73—C74—C75	0.8 (5)
C17—C18—C19—C20	177.8 (4)	C71—N3—C75—C74	0.0 (5)
C18—C19—C20—C21	−0.2 (6)	Zn4—N3—C75—C74	−178.4 (3)
C19—C20—C21—C22	0.7 (6)	C73—C74—C75—N3	−0.3 (5)
C19—C20—C21—C24	−179.8 (4)	C71—C72—C76—O20	166.0 (3)
C20—C21—C22—C23	−0.8 (6)	C73—C72—C76—O20	−11.1 (5)
C24—C21—C22—C23	179.6 (4)	C71—C72—C76—N4	−14.5 (5)
C21—C22—C23—C18	0.4 (6)	C73—C72—C76—N4	168.4 (3)
C19—C18—C23—C22	0.1 (6)		

### Hydrogen-bond geometry (Å, °)

**Table:**

*D*—H···*A*	*D*—H	H···*A*	*D*···*A*	*D*—H···*A*
N2—H2A···O5^i^	0.88	2.29	3.161 (4)	173
N2—H2B···O21	0.88	2.06	2.918 (4)	165
N4—H4A···O7^ii^	0.88	2.19	3.061 (4)	168
N4—H4B···O22	0.88	2.08	2.939 (5)	166
O9—H91···O5^iii^	0.97	1.65	2.602 (3)	166
O9—H92···O19^iv^	0.97	1.68	2.644 (4)	172
O10—H101···O20^v^	0.97	1.67	2.639 (4)	176
O10—H102···O7^vi^	0.97	1.78	2.649 (3)	147
O21—H211···O15	0.97	1.94	2.869 (4)	159
O21—H212···O19^vii^	0.86	2.29	3.138 (4)	171
O22—H221···O20^viii^	0.97	2.23	3.164 (4)	160
O22—H222···O18	0.97	1.90	2.802 (5)	152
C65—H65···O21	0.95	2.37	3.282 (4)	161
C71—H71···O22	0.95	2.33	3.256 (5)	165

Symmetry codes: (i) *x*, *y*+1, *z*; (ii) *x*+1, *y*+1, *z*; (iii) *x*, −*y*, *z*−1/2; (iv) *x*, −*y*+1, *z*−1/2; (v) *x*−1, −*y*+1, *z*+1/2; (vi) *x*, −*y*, *z*+1/2; (vii) *x*, −*y*+2, *z*−1/2; (viii) *x*, −*y*+2, *z*+1/2.

## References

[bb1] Bruker (2005). *SADABS* Bruker AXS Inc., Madison, Wisconsin, USA.

[bb2] Bruker (2007). *APEX2* and *SAINT* Bruker AXS Inc., Madison, Wisconsin, USA.

[bb3] Farrugia, L. J. (1999). *J. Appl. Cryst.* **32**, 837–838.

[bb4] Flack, H. D. (1983). *Acta Cryst.* A**39**, 876–881.

[bb5] Greenaway, F. T., Pezeshk, A., Cordes, A. W., Noble, M. C. & Sorenson, J. R. J. (1984). *Inorg. Chim. Acta*, **93**, 67–71.

[bb6] Hökelek, T., Dal, H., Tercan, B., Aybirdi, Ö. & Necefoğlu, H. (2009*b*). *Acta Cryst.* E**65**, m627–m628. 10.1107/S1600536809016602 PMC2969611 21582996

[bb7] Hökelek, T., Dal, H., Tercan, B., Aybirdi, Ö. & Necefoğlu, H. (2009*c*). *Acta Cryst.* E**65**, m1037–m1038. 10.1107/S1600536809027093 PMC2970058 21577401

[bb8] Hökelek, T., Dal, H., Tercan, B., Aybirdi, Ö. & Necefoğlu, H. (2009*d*). *Acta Cryst.* E**65**, m1365–m1366. 10.1107/S1600536809041208 PMC2970957 21578119

[bb9] Hökelek, T. & Necefoğlu, H. (1996). *Acta Cryst.* C**52**, 1128–1131.

[bb10] Hökelek, T., Yılmaz, F., Tercan, B., Gürgen, F. & Necefoğlu, H. (2009*a*). *Acta Cryst.* E**65**, m1416–m1417. 10.1107/S1600536809042640 PMC2971022 21578154

[bb11] Macrae, C. F., Edgington, P. R., McCabe, P., Pidcock, E., Shields, G. P., Taylor, R., Towler, M. & van de Streek, J. (2006). *J. Appl. Cryst.* **39**, 453–457.

[bb12] Sheldrick, G. M. (2008). *Acta Cryst.* A**64**, 112–122. 10.1107/S0108767307043930 18156677

[bb13] Spek, A. L. (2009). *Acta Cryst.* D**65**, 148–155. 10.1107/S090744490804362X PMC2631630 19171970

